# Right pneumothorax, pneumomediastinum, pneumoperitoneum, pneumoretroperitoneum, and subcutaneous emphysema as rare complications after ERCP: a case report

**DOI:** 10.1093/omcr/omae118

**Published:** 2024-09-22

**Authors:** Ghassan Bayat, Farah Haneyah, Laura Merjaneh, Sultaneh Haddad, Aboud Thaljah, Jack Zambakjian, Mike Ghabally

**Affiliations:** Plymouth University Hospitals NHS Trust, Plymouth, United Kingdom; Al-balqa Applied University, Hashemite Kingdom of Jordan; Division of Gastroenterology, Department of Internal Medicine, Zahi Azrak Hospital, Aleppo, Syrian Arab Republic; Division of Pediatric, Children’s Hospital, Damascus, Syrian Arab Republic; Stemosis for Scientific Research, Damascus, Syrian Arab Republic; Division of Gastroenterology, Department of Internal Medicine, Faculty of Medicine, University of Aleppo, Aleppo, Syrian Arab Republic; Division of Gastroenterology, Department of Internal Medicine, Faculty of Medicine, University of Aleppo, Aleppo, Syrian Arab Republic; Division of Cardiology, Department of Internal Medicine, Faculty of Medicine, University of Aleppo, Aleppo, Syria

**Keywords:** ERCP, pneumothorax, pneumomediastinum, pneumoperitoneum, subcutaneous emphysema

## Abstract

Endoscopic retrograde cholangiopancreatography is a complex procedure with a significant risk of severe consequences. We herein report a 56-year-old Middle Eastern female who was diagnosed with acute ascending cholangitis. Endoscopic retrograde cholangiopancreatography was performed with gallstone absorption and stent implanting. However, the patient developed significant pneumothorax; pneumomediastinum, pneumoperitoneum, pneumoretroperitoneum, and subcutaneous emphysema of the abdomen, chest, right arm and shoulder, face and right orbital area. Radiological studies demonstrated no evidence of perforation on bowel obstruction. The patient was treated successfully with good results and post-operative follow-up was unremarkable. In conclusion, air leakage following endoscopic retrograde cholangiopancreatography without evidence of perforation is extremely rare. While pneumothorax development usually requires thoracostomy; pneumomediastinum, pneumoperitoneum, pneumoretroperitoneum, and subcutaneous emphysema are usually treated conservatively.

## Introduction

Endoscopic retrograde cholangiopancreatography (ERCP) is a complex, technically demanding procedure with a significant risk of severe consequences. According to reports, complication rates of ERCP range from 5 to 6.9% including pancreatitis, hemorrhage, sepsis, and perforation with a mortality rate of 0.33% [1]. Post-ERCP pneumothorax, pneumomediastinum, pneumoperitoneum, subcutaneous emphysema, and pneumoretroperitoneum, are extremely rare complications of ERCP in the medical literature. For instance, there is only one documented case of bilateral pneumothorax. A duodenal puncture is the most common source of air leakage. Nonetheless, if there isn’t a visible hole, the usage of compressed air to keep a lumen patent is most likely connected to air dissection [2].

We believe that awareness of these potential complications should be raised regarding possible etiologies, therapeutic principles, and prognosis, particularly among gastroenterologists, as it is an uncommon, unexpected, and potentially life-threatening event [1].

## Case presentation

A 56-year-old Middle Eastern female presented to our emergency department with a chief complaint of abdominal pain associated with nausea, fever, chills, and progressive jaundice over the last three days. The abdominal pain started as an epigastric colic pain that transferred into the right upper quadrant, radiating to the right shoulder and exacerbating with fatty meals. The fever was intermittent and ranged between 38.5–39.5°C. Her past medical history included well-controlled hypertension, type-2 diabetes mellitus, and dyslipidemia. The surgical and family history is unremarkable. Her medications included valsartan, amlodipine, metformin, and atorvastatin.

On examination, her vital signs were stable and she had overt jaundice in her sclera and skin. Abdominal examination demonstrated epigastric and right upper quadrant tenderness with a positive Murphy’s sign. Laboratory workup was consistent with the clinical examination, as follows: Hemoglobin: 10.3 mg/dL, WBC: 4.160 × 109/l, PLT: 109 000 × 109/l, Urea: 37 mg/dl, Creatinine: 1.1 mg/dl, SGPT: 110 mg/dl, SGOT: 263 mg/dl, Total Bilirubin: 7.2 mg/dl (direct: 6.2 mg/dl, indirect: 0.9 mg/dl), Alkaline Phosphatase: 517 mg/dl, C-reactive protein: 14.5 mg/dl, albumin: 3.8 g/dl, cholesterol: 148 mg/dl, potassium: 3.7 mEq/l, sodium: 148 mEq/l.

Abdominal ultrasound demonstrated a non-edematous gallbladder measuring 10.5×4.4 cm and containing dense sludge without obvious gallstones. The common bile duct was dilated (13 mm), as well as the intrahepatic ducts. However, a detailed examination of the portal vein, pancreas, and other abdominal organs was completely normal. Thus, the patient was diagnosed with acute ascending cholangitis. The patient was admitted, and intravenous antibiotics were initiated as well as supportive therapy.

**Figure 1:**
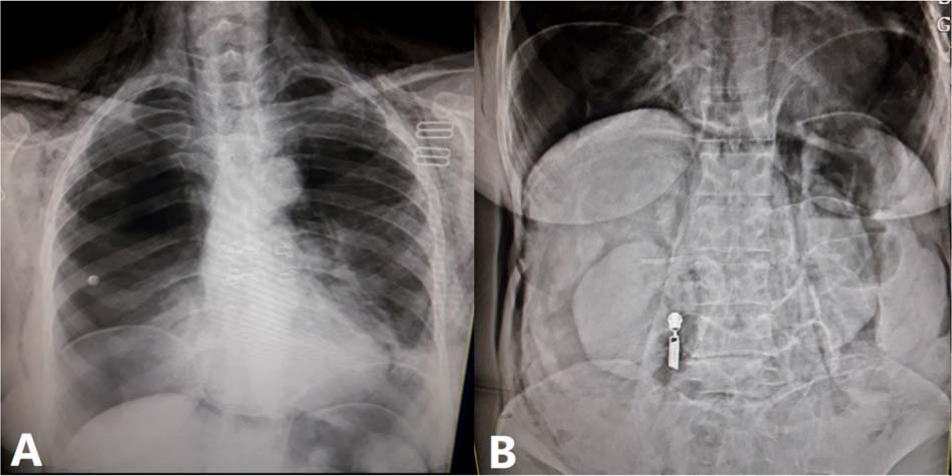
Chest X-ray. (**A**) Represents a chest X-ray revealing the presence of right pneumothorax with right lung atelectasis, pneumopericardium, and pneumoretroperitoneum. (**B**) Abdominal X-ray demonstrating the presence of pneumoperitoneum and pneumoretroperitoneum.

Endoscopic Retrograde Cholangiopancreatography (ERCP) under sedation with propofol without intubation was performed within 24 h and air was used during the procedure. The duodenum appeared small with endoscopic alteration as the Ampulla of Vater originated backward. A papillectomy could not be performed despite multiple attempts; thus, a knife precut was done. The common bile duct was subsequently cannulated and appeared dilated before obstruction at its end. A 10.5 mm gallstone was extracted and an 8 French wide, 10 cm long plastic stent was implanted, resulting in good bile drainage without any intraoperative complications. The procedure was difficult and prolonged lasting almost 60 min. Unfortunately, the patient started to develop dyspnea over 5 min (SpO2 98% → 90% without oxygen, 96% with oxygen; respiratory rate 16 → 31), and she woke up agitated. Furthermore, characteristic progressive subcutaneous crepitations developed over the right side of the abdomen, chest, right shoulder, neck, right side of the face, and right orbital cavity. Abdominal examination revealed moderate abdominal distention and hyperresonance to percussion, without tenderness or rebound tenderness. Lung auscultation revealed notably decreased respiratory sounds in the middle and upper portions of the right lung. However, there were no signs of hemodynamic instability, and thus, abdominal and chest X-rays were performed demonstrating the presence of right pneumothorax, pneumomediastinum, pneumoperitoneum, and subcutaneous emphysema (Fig. 1). Consequently, a chest tube was placed for the right pneumothorax, leading to symptomatic relief as well as vital signs’ improvement (SpO2 98% without oxygen and the respiratory rate dropped to 18). In the absence of surgical abdominal conditions, peritoneal pneumothorax was treated conservatively with antibiotics, intravenous fluids, and bowel rest.

**Figure 2:**
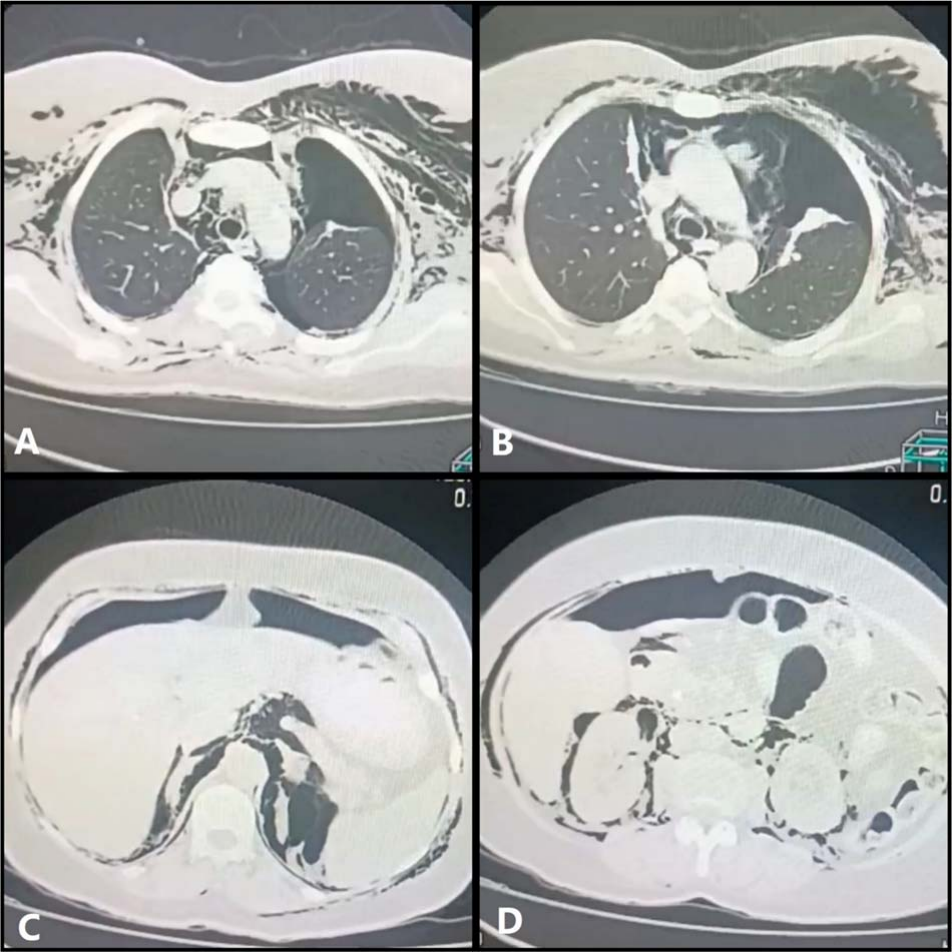
Computerized Tomography with contrast substance ingestion of the chest, abdomen, and pelvis. (**A** and **B**) Reveal the presence of left pneumothorax, pneumopericardium, and subcutaneous emphysema with a right-sided chest tube with good lung expansion. (**C** and **D**) Demonstrates the presence of pneumopericardium and pneumoretroperitoneum.

On the second day, mild to moderate dyspnea recurred with decreased respiratory sounds in the left lung field with hyperresonance to percussion. A computerized tomography (CT) scan of the chest, abdomen, and pelvis with contrast substance ingestion was performed, revealing the presence of a left pneumothorax, good expansion of the right lung, pneumomediastinum, pneumoperitoneum, pneumoretroperitoneum and subcutaneous emphysema without extravasation of contrast materials or any surgical complications (Fig. 2). Consequently, a left chest tube was placed, resulting in total relief of dyspnea. The patient’s condition started to gradually improve, and the chest tubes were removed on the 5th day. Moreover, the subcutaneous emphysema improved over 1 week as well. As a result, the patient was discharged on the seventh day in good condition. The patient was followed up every week for 1 month with a detailed physical examination and repeated chest X-rays. Subsequently, a laparoscopic cholecystectomy was performed, and the common bile duct stent was removed after 1 week. Additionally, the 90-day post-operative follow-up was unremarkable.

## Discussion

Post-ERCP pneumomediastinum, pneumothorax, and subcutaneous emphysema without evidence of perforation are extremely rare entities in the medical literature. The main risk factors are cirrhosis, difficult bile duct cannulation, suspected sphincter of Oddi dysfunction, previous gastrointestinal surgery, and pre-cut technique [3]. According to the Stapfer classification, our case is classified as type four as retroperitoneal air is alone.

Four possible routes to the mediastinal and pleural spaces: (1) in a retroperitoneal perforation, air can leak throughout the continuous fascial planes, to the thoracic cavity (2) in a free peritoneal perforation, air can communicate between the thoracic and abdominal cavities through diaphragmatic orifices (3) in the absence of perforation, air can enter the mucosa through a weak spot (e.g. anastomosis, diverticulum) (4) finally, air from a ruptured alveolus (through hyperventilation, the Valsalva maneuver,) can track into the perivascular sheath, which could cause pulmonary interstitial emphysema [4]. To identify the tension pneumoperitoneum we measured the pressure inside the bladder, which was less than 20 mm so tension pneumoperitoneum did not exist in our case.

Initially, we used the lowest risk and most familiar cannulation technique; by advancing past the junction of the first and second part of the duodenum before scope rotating and shortening (by sideway angulation to the right followed by upward angulation of the tip and rotating the left wrist to the right while pulling back the scope gently). Markings on the duodenoscope indicated 60–65 cm at the incisors. In patients with normal anatomy, the papilla is usually well visualized on the posteromedial wall of the second portion of the duodenum; however, in our patient, the papilla was far from the scope tip (located more posteriorly) while advancing the scope or performing the shortening maneuver. Contractions were reduced by using intravenous injection of hyoscine butyl bromide. We didn’t get the optimal axis of the distal bile duct nor the optimal orientation, and the alignment of the accessories in line with the axis of the distal bile duct was poor, hence, we failed cannulation after many attempts and we decided to use needle-knife (The papilla was incised from “below-upward” starting from the papillary orifice and cutting up toward the 11 o’clock direction). The bile duct was then selectively cannulated using a soft guidewire through the needle knife.

There are several reported causes of subcutaneous emphysema in the literature. There have been correlations shown between spontaneous or traumatic pneumothorax, iatrogenic or traumatic tracheal injury, and facial trauma and sinus injury. The implantation of a gastrostomy tube and laparoscopic surgery can also occasionally result in subcutaneous emphysema. Another known etiology is perforation of the gastrointestinal system during an endoscopic operation [5].

Alterations in physiological parameters such as vital signs, the onset of dyspnea, and a decline in oxygen saturation, particularly after pre-cut sphincterotomy, warrant consideration of potential retroperitoneal perforation or pneumothorax. In the majority of instances involving retroperitoneal air and pneumothorax, additional imaging beyond chest and plain abdominal X-rays may not be necessary. However, in cases where these conditions are not readily apparent and a patient’s clinical condition deteriorates following ERCP, displaying symptoms and signs indicative of a potentially serious complication, abdominal and/or chest CT scanning is warranted [1].

Currently, there is a lack of established guidelines or a consensus regarding the management of duodenal perforations. The approach to perforation management is determined by factors such as the type of perforation, radiological findings, the severity of the injury, the patient’s condition, and the physician’s expertise. Given the presence of factors that render surgery less favorable for certain patient demographics, such as older adults, individuals with frailty, and those with comorbidities like longstanding hypertension, conservative treatment may emerge as the preferred course of action [6].

Although surgical treatment is required for almost 25% of cases, mortality rates of ERCP-related pneumothorax are less than 10% of all cases [7].

Our patient started to develop dyspnea after ERCP and she doesn’t have a previous tumor or anatomical deformity according to an earlier tumor so we do not need to do pre-ERCP ultrasound and CT/MRCP.

Furthermore, characteristic progressive subcutaneous crepitations developed over the right side of the abdomen, chest, right shoulder, neck, right side of the face, and right orbital cavity. The presence of risk factors such as previous gastrointestinal surgery, difficult and prolonged procedure, and pre-cut technique, we were prompted to suspect that these complications, which were confirmed through radiographic imaging were related to ERCP. Our patient responded exceptionally well to conservative treatment.

## Conclusion

Pneumothorax represents a seldom encountered yet alarming complication arising from ERCP procedures. The predominant pathophysiological mechanism entails retroperitoneal perforation stemming from pre-cut sphincterotomy, subsequently leading to the onset of pneumomediastinum and subsequent pneumothorax. Employing conservative management strategies, including pleural drainage when necessary, typically results in prompt and comprehensive patient recovery.

## Consent

Written informed consent was obtained from the patient to publish this case report and accompanying images. A copy of the written consent is available for review by the Editor-in-Chief of this journal on request”.

## Guarantor

All authors have read and approved the manuscript, on behalf of all the contributors I will act and guarantor and will correspond with the journal from this point onward.
